# Supplementary material for: Allergenic Shrimp Tropomyosin Distinguishes from a Non-Allergenic Chicken Homolog by Pronounced Intestinal Barrier Disruption and Downstream Th2 Responses in Epithelial and Dendritic Cell (Co)Culture
Source: Nutrients. 2024 Apr 17;16(8):1192. doi: 10.3390/nu16081192 (PMC11053543; doi:10.3390/nu16081192)
Supplement: Supplementary file 1 [file nutrients-16-01192-s001.zip › nutrients-2931793-supplementary.pdf]

Supplementary Figures

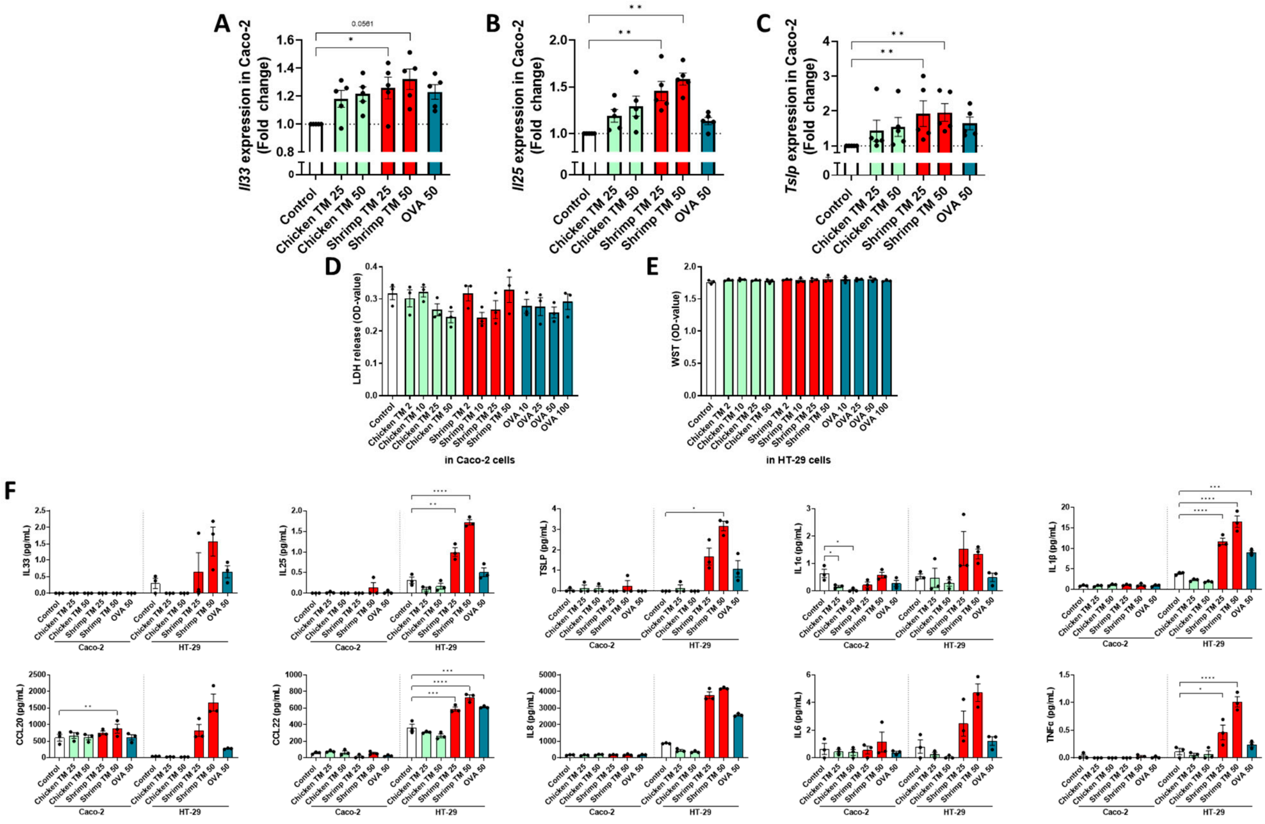

Figure S1. Fold change in gene expression was measured 8h after 25 or 50 µg/mL chicken TM, shrimp TM or OVA exposure in Caco-2 cells cocultured with PBMCs. mRNA expression of the alarmins (A) Il33, (B) Il25, and (C) Tslp was increased upon exposure to either concentration of sshrimp TM. D) LDH release and (E) WST conversion by Caco-2 cells and HT-29 cells respectively was not affected 48h after exposure in 96 wells flat bottom plates to increasing doses of chicken TM, shrimp TM or OVA. (F) A multiplex array was performed on supernatants from Caco-2 cells and HT-29 cells that were exposed to 25 and 50 µg/mL chicken TM or shrimp TM, or 50 µg/mL OVA. Next to the increased secretion of IL25, TSLP, IL1β, CCL22, and TNFα after exposing HT-29 cells to 50 µg/mL sdhrimp TM, a similar effect on cytokine secretion was seen after exposure to 25 µg/mL shrimp TM. Both chicken TM exposures decreased secretion of IL1α in Caco-2 cells, but exposure to this low-allergenic protein did not induce any other changes in cytokines secretion from Caco-2 cells or HT-29 cells. Data is analyzed by One-Way ANOVA or Friedman test when data did not fit a normal distribution, n=3 or n=5, mean ± SEM (* $p < 0.05$, ** $p < 0.01$, *** $p < 0.001$, **** $p < 0.0001$).

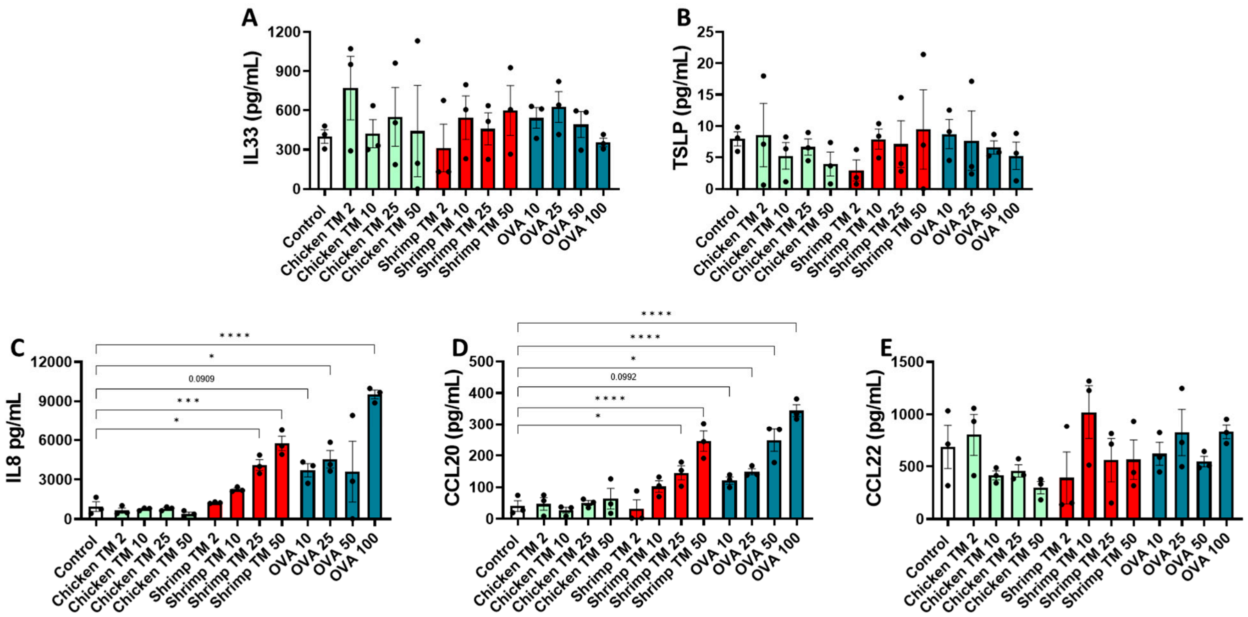

Figure S2. HT-29 cells were exposed to increasing doses of chicken TM, shrimp TM or OVA for 48h in 96 wells flat bottom plates. Secreted A) IL33, B) TSLP, C) IL8, D) CCL20, and E) CCL22 were measured by ELISA. Exposure to chicken TM did not enhance cytokine secretion, exposure to shrimp TM and OVA induced a dose-dependent enhanced secretion of IL8 and CCL20. Data is analyzed by One-Way ANOVA, n=3, mean ± SEM (* $p < 0.05$, ** $p < 0.01$, *** $p < 0.001$, **** $p < 0.0001$).

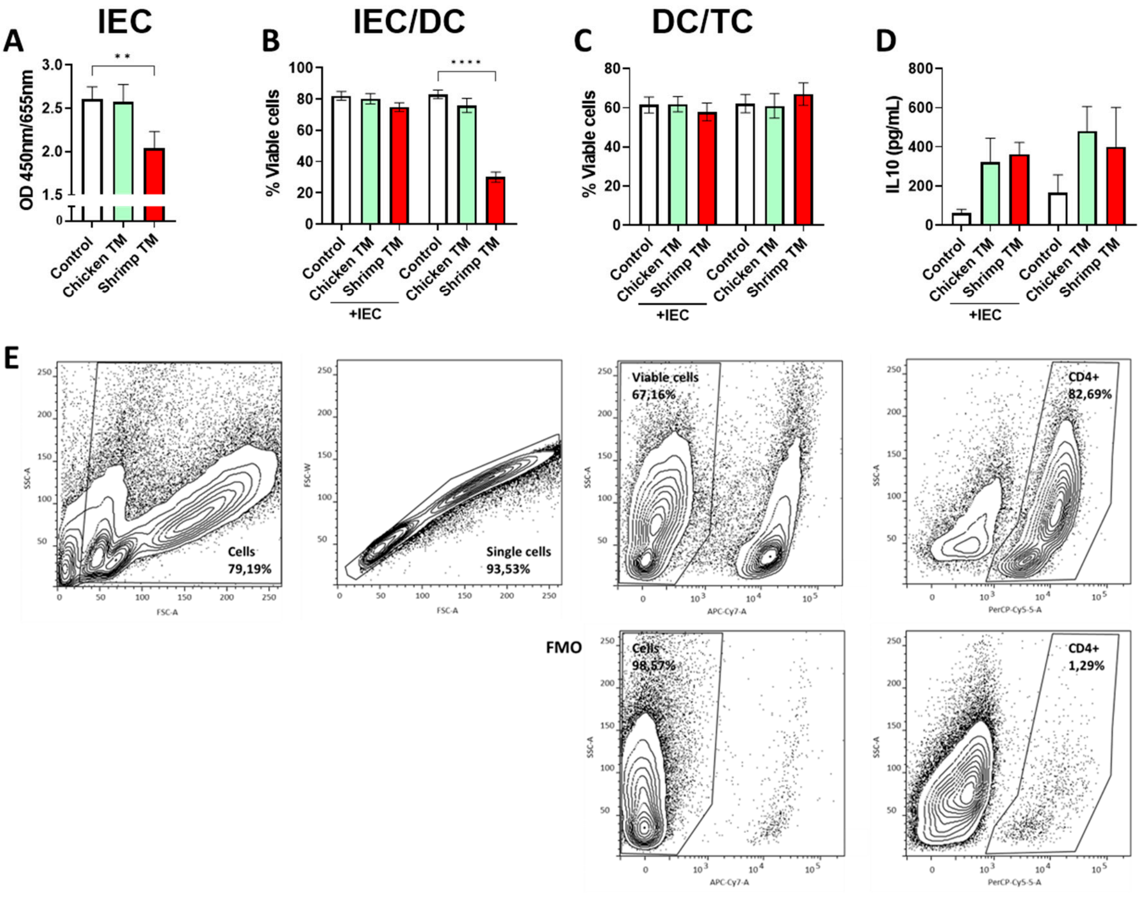

Figure S3. After culture of moDC with or without HT-29 cells and coculture of primed DC with T cells, viability was assessed. Viability of A) HT-29 cells after 50 µg/mL shrimp TM exposure. B) Viability of moDC was not affected by chicken TM or shrimp TM exposure when cocultured with HT-29 cells. However when moDCs were directly exposed to TmH, cell viability was significantly

decreased. Coculture of the primed DCs with T cells did not affect C) viability or D) IL10 secretion. E) Furthermore, the gating strategy used to determine the viable T helper cells population and corresponding FMOs are presented. Data is analyzed by One-Way ANOVA, n = 3, mean ± SEM (* $p < 0.05$, ** $p < 0.01$, *** $p < 0.001$, **** $p < 0.0001$).

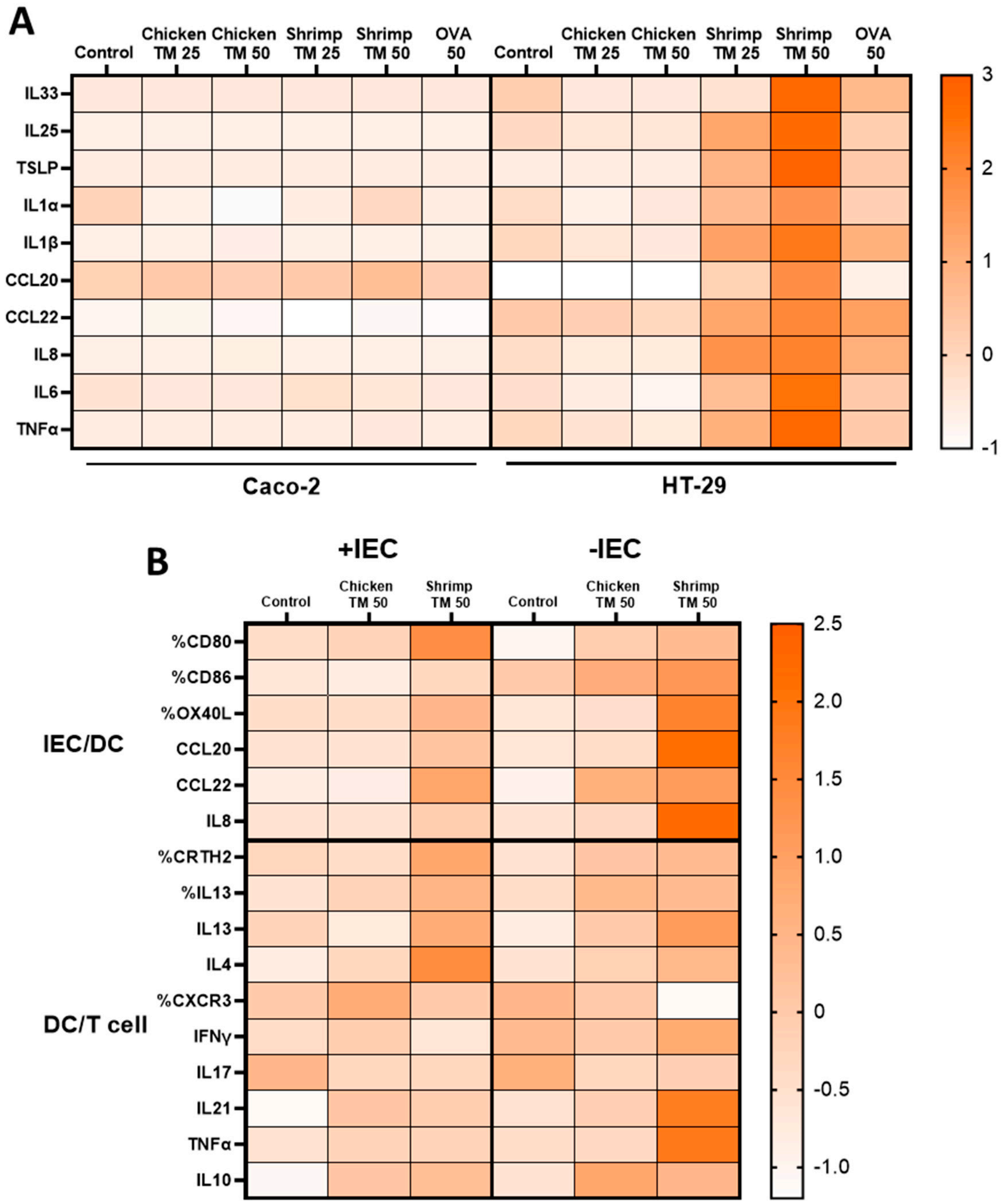

Figure S4. A visual representation, using z-scores, of (A) the cytokine secretion from Caco-2 and HT29 cells cultured in 96 well flatbottom culture plates and, (B) cytokine secretion and marker expression upon exposure to chicken TM or shrimp TM in the IEC/DC/T cell coculture model.

10 20 30 40 50 60

MDAIKKKMQM LKLDKENALD RAEQAEADKK AAEERSKQLE DELVALQKKL KGTEDELDKY

70 80 90 100 110 120

SESLKDAQEK LELADKKATD AESEVASLNR RIQLVEEELD RAQERLATAL QKLEEAEKAA

130 140 150 160 170 180

DESERGMKVI ENRAQKDEEK MEIQEIQLKE AKHIAEEADR KYEEVARKLV IIEGDLERAE

190 200 210 220 230 240

ERAELSESKC AELEEELKTV TNNLKSLEAQ AEKYSQKEDK YEEEIKVLTD KLKEAETRAE

250 260 270 280 290

FAERSVTKLE KSIDDLEDEL YAQKLKYKAI SEELDHALND MTSI*AAALE***H HHHHH**

Figure S5. Amino acid (aa) sequence of recombinant chicken α-1 tropomyosin (UniProt P04268), including residual aa from cloning site (italic) and His6-Tag (bold), with a calculated molecular weight of 34.044 kDa and pI of 4.83 (Expasy ProtParam).

10 20 30 40 50 60

M*RGS*DAIKKK MQAMKLEKDN AMDRADTLEQ QNKEANNRAE KSEEEVHNLQ KRMQQLENDL

70 80 90 100 110 120

DQVQESLLKA NIQLVEKDKA LSNAEGEVAA LNRRIQLLEE DLERSEERLN TATTKLAEAS

130 140 150 160 170 180

QAADESERMR KVLENRSLSD EERMDALENQ LKEARFLAEE ADRKYDEVAR KLAMVEADLE

190 200 210 220 230 240

RAEERAETGE SKIVELEEEL RVVGNNLKSL EVSEEKANQR EEAYKEQIKT LTNKLKAAEA

250 260 270 280 290

RAEFAERSVQ KLQKEVDRLE DELVNEKEKY KSITDELDQT FSELSGYRS**H HHHHH**

Figure S6. Amino acid (aa) sequence of recombinant shrimp tropomyosin (UniProt A1KYZ2), including residual aa from cloning site (italic) and His6-Tag (bold), with a calculated molecular weight of 34.2 kDa and pI of 4.96 (Expasy ProtParam).

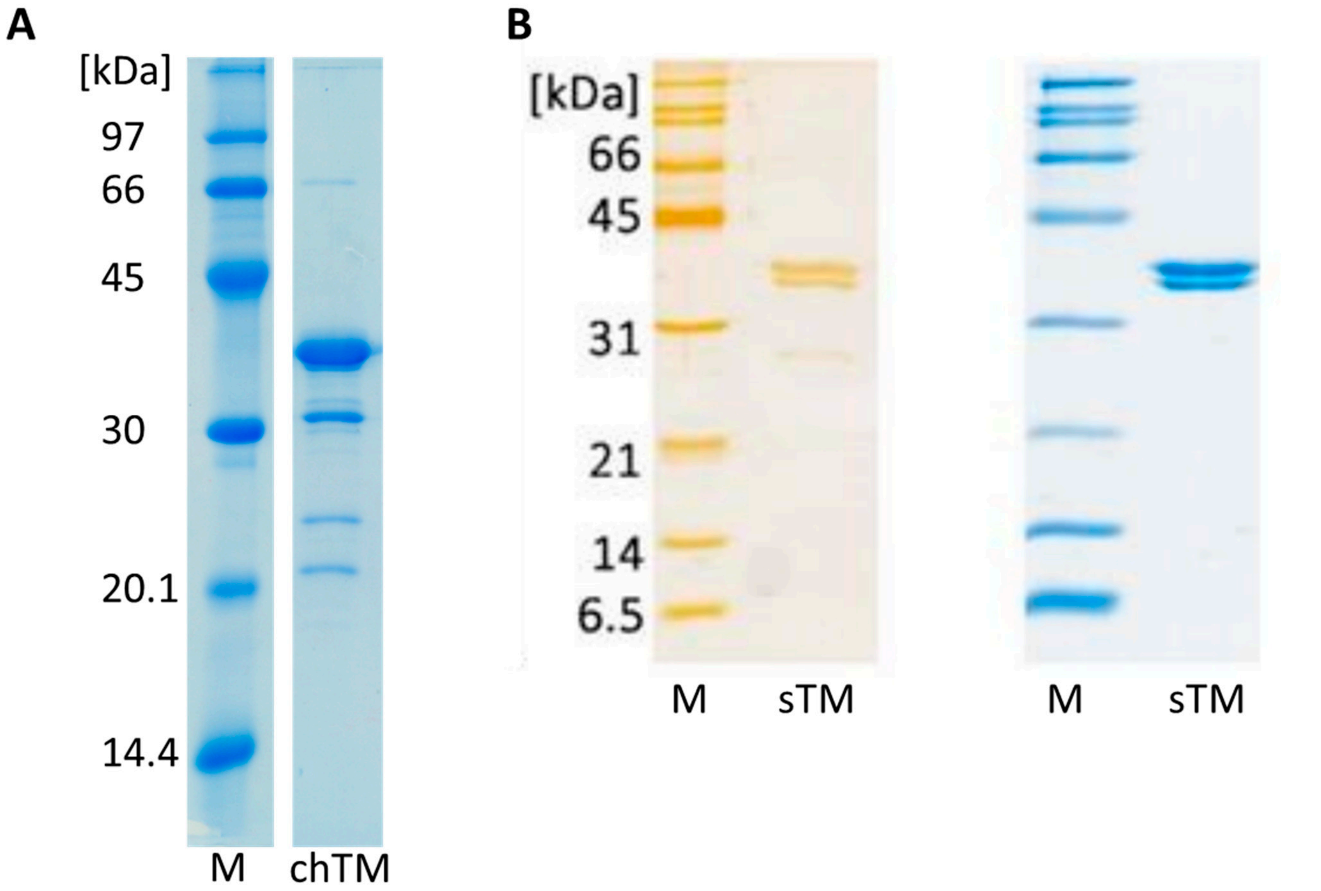

Figure S7. (A) Coomassie-stained chicken tropomyosin after removal of endotoxin with a major protein band around 36 kDa. (B) Silver stained and Coomassie stained shrimp tropomyosin after removal of endotoxin with a major protein band around 35 kDa (M, molecular kDa marker; chTM, chicken tropomyosin; sTM, shrimp tropomyosin).

Supplementary Methods

1.1. Cloning and Expression of Recombinant Chicken Tropomyosin α-1 Chain Isoform X1

Recombinant chicken tropomyosin α-1 chain isoform X1 (UniProt acc. no. P04268) was expressed in *E coli* BL21 (DE3) with a C-terminal His_6-tag, as described previously (1), purified by immobilized metal affinity chromatography (IMAC) (2), and anion exchange chromatogtaphy (AEC) according to manufacturer's instructions.

Briefly, purchased tropomyosin DNA string (GeneArt, Thermo Fisher Scientific, Germany) was cloned with a C-terminal His_6-tag using vector pET23b (Novagen/Merck, Germany) and in-Fusion ecodry cloning Kit (Clontech, Takara Bio, USA). Competent E. coli BL21 (DE3) pLysS (Stratagene/Agilent Technologies, Germany) cells were transformed, and expression was chemically induced by adding isopropyl-beta-D-thiogalactopyranoside (IPTG). After lysis in a cell disruptor (Constant Systems Limited, Low March, UK) and centrifugation, recombinant chicken tropomyosin in supernatant was filtered through 0.45 µm asymmetrical polyethersulfone membrane (Thermo Fisher Scientific) and added to Ni-NTA Superflow bead (Qiagen, Germany) in equilibration buffer (20 mM Tris, pH 8, 10 mM NaCL), and incubated overnight at 4°C. After packing into an ECO10/120VOV column (YMC Europe GmbH, Germany) and washing in equilibration buffer (80 min at 0.3 mL/min flow rate), tropomyosin was eluted within 80 min, using 500 mM imidazol in equilibration buffer (0.3 mL/min flow rate). This was followed by subsequent purification by AEC on HiTrap Capto Q ImpRes column (Cytiva Europe, Germany) according to manufacturer's instructions. Both chromatography steps were run on an ÄKTA pure 25 M (Cytiva Europe, Germany). Purified chicken tropomyosin was dialyzed against low salt

buffer (20 mM Tris, pH 8, 10 mM NaCl, 1 mM EDTA) in D-Tube Dialyzer Maxi 3.5 kDa molecular weight cutoff (Merck, Darmstadt, Germany) for further analysis.

1.2. Cloning and Expression of Recombinant Shrimp Tropomyosin

The shrimp tropomyosin gene, the gene encoding Pen m 1, was published by Motoyama *et al.* (3). The corresponding cDNA (A1KYZ2) was ordered at Eurofins (Ebersberg, Germany) in the cloning vector pEX-A2. Specific Bam HI- and BgIII-sites were introduced for later subcloning into vector pQE-16. Transformation of TOP10 cells was done with pEX-A2-shrimp TM plasmid. TOP 10 cell clones were screened for the presence of pEX-A2-shrimp TM plasmid. The pEX-A2-shrimp plasmid was purified, subjected to a double digestion done using BamHI and BgIII and ligated into pQE-16. This new plasmid was transformed/secured into E.coli XL1-Blue cells. After DNA sequencing control, the pQE-16-shrimp TM plasmid was transformed into expression host E.coli M15. In production culture, the protein expression was induced using IPTG.

Bacterial pellets were lysed by resuspension with 12 mL lysis buffer (20 mM KH2PO4, 0.5 M NaCl, 10 mM Imidazole, pH 8) and sonicated twice by Biologics Model 150 VT Ultrasonic Homogenizer for 2 min, 40% pulse mode with 50% power on ice. Cells were incubated with lysozyme 1 mg/mL (Sigma, 62970) and benzonase (Merck, 101654) 1250 U per 0.6 L for 30 min on a tube rotator. After centrifugation, supernatants were filtered by Millex 0.22 µm and loaded on Profinia His-Trap (Biorad).

Recombinant protein was purified using a PROFINIA His-Trap device with a Protino® Ni-NTA Column 1 mL (MACHEREY-NAGEL, 745410.1), a purification system used to perform an immobilized metal affinity chromatography (IMAC). Recombinant Pen m 1 was eluted with 500 mM imidazol-containing buffer. Buffer exchange to PBS buffer was performed with fractions containing recombinant protein.

1.3. Physicochemical Confirmation and Characterization of Recombinant Tropomyosins

1.3.1. Chicken TM

The identity of recombinant chicken tropomyosin α-1 chain was analyzed by mass spectrometry (MS) as described earlier for natural pea 2S albumin nPA1 on a Synapt G2-Si (2). Differing from this, MS^E data were searched against an in-house, UniProt (as of 2016) derived database consisting of reviewed entries of all species and the amino acid sequence of the recombinant tropomyosin (Supplemental Figure S5).

Secondary structure elements were analyzed using UV-circular dichroism (CD) spectroscopy (Jasco J-810S, Jasco Germany GmbH, Pfungstadt, Germany) in low salt buffer. Hydrodynamic radii (R_H) were determined by dynamic light scattering (DLS, Zetasizer Nano-127 ZS, software v6.12, Malvern Intruments GmbH, Herrenberg, Germany) in low salt buffer.

1.3.2. Shrimp TM

The protein identity was verified by MS analyses (amino acid sequence is shown in Supplemental Figure S6). Trypsin-digested recombinant tropomyosin (Pen m 1) was spotted on a MALDI plate (Polished steel 384 MALDI target plate, Bruker) and 0.3 µl of matrix solution added (5 mg/mL alpha-cyano-4-hydroxycinnamic acid, HCCA, Bruker and 1 mg/mL 2,5-dihydroxybenoiquem DHB, Bruker in 50% Acetonitile containing 0.1% TFA) according to dried-droplet method. An external calibration was done before each analysis with trypsin digested bovine serum albumin (Brucker manufacturer's instructions). Protein mass finger print (PMF) was generated and compared to in silico digestions of TM in NCBInr database (Mascot server, Matrix Science). Confirmatory Edman sequencing was performed on a Procise 49X HT protein sequencer (Applied Biosystems). CD was used for studying secondary protein structures. Samples were measure in a cuvette of 0.1 cm path length using the Chirascan CD spectrometer (Applied Photophysics). Far-Ultraviolet CD spectra were recorded at 20°C starting with a wavelength of 180 nm to 260 nm (1 nm bandwidth, 0.5 seconds interval, 5 repeats). The read-out was converted with respective

protein details into degrees*cm2*dmol–1 according to the manufacturer's instructions Dichroweb was used to analyze circular dichroism data.

1.4. Endotoxin removal and concentration determination

1.4.1. Chicken TM

After four cycles of endotoxin removal using EndoTrap red-kit (Lionex GmbH, Braunschweig, Germany) according to manufacturer's instructions, the final preparation of recombinant chicken tropomyosin and recombinant shrimp tropomyosin was gained in equilibration buffer (phosphate buffer, pH 7.4, 80 mM NaCl), and endotoxin was quantified using the LAL kinetic turbidimetric assay according to Remillard *et al.*(4), resulting to 0,809 EU/mg endotoxin. Protein was quantified against bovine serum albumin dilution series by densitometry (ImageJ) of Coomassie-stained SDS-PAGE gel.

1.4.2. Shrimp TM

The removal of endotoxins was achieved by endotoxin removal columns EndoTrap red 5/1 (Endotrap, Hyglos, Germany). The protein was eluted with 10 mM Na2HPO4, 80 mM NaCl, pH 7.4 and tested for endotoxin content according to the protocol of the Pierce LAL chromogenic endotoxin quantification kit (Thermo Scientific, Rockford, US), resulting to 165 EU/mg endotoxin. Protein was quantified by the Bradford method (Biorad, Nazareth, Belgium) using bovine serum albumin (Sigma, US) as standard protein (0.001-0.008 mg/ml) by measuring the absorption at 595 nm using a spectrophotometer (Ultraspec III, Pharmacia LKB).

1.5. Allergen preparations

1.5.1. Chicken TM

In Coomassie-stained SDS-PAGE, the purified recombinant His_6-tagged chicken α-1 tropomyosin appeared as a major protein band (estimated 90 % of total protein) around 36 kDa and according to a calculated molecular mass of 34.044 kDa (Supplemental Figure S7A), and comparable to the work previously published (1).

Purified recombinant chicken tropomyosin showed a typical α-helical signature (maximum at 195 nm, minima at 208 nm and 222 nm) in UV CD-spectroscopy, which was comparable to the data previously published (1). In DLS analysis, a mean R_H of 7.76 (+ 0.76) nm indicated some level of aggregation of the major peak (> 60 % mass) that was interpreted as monodisperse.

Sequence identity of His_6-tagged recombinant chicken tropomyosin (based on UniProt Acc. No. P04268) was confirmed with a protein score of 18798 by detecting 130 peptides with a mean mass error of 2.0 ppm, covering 58.0 % of the amino acid sequence (Supplemental Figure S5). Chicken Tropomyosin was finally gained at 346 µg/mL in phosphate buffered saline (pH 7.4, 80 mM NaCl). The amount of residual LPS in recombinant chicken tropomyosin was quantified at 0.28 EU/mL after repetitive LPS removal.

1.5.1.1. Shrimp TM

To verify the purity of Pen m 1, a SDS-PAGE followed by silver stain was performed. A tropomyosin-like double band was visualised at 35 kDa (Supplemental Figure S7B). To summarize, a total amount of 2.5 mg pure, recombinant tiger prawn tropomyosin (Pen m 1) was made available.

MS-based analyses using PMF gave a sequence coverage of 91% sequence coverage to the previously in the laboratory cloned and translated cDNA sequence of tropomyosin of giant tiger prawn (database no. A1KYZ2).

Using CD analyses, recombinant shrimp TM showed an alpha-helical folded, reflected by typical curves with two characteristic minima at 208 nm and 222 nm. The ratio of the negative peak intensity '222 nm/208 nm' was 1.081 for recombinant Pen m 1, indicating that the TM had characteristic coil-coil structures. Using the Dichroweb tool, the shrimp TM had an alpha-helical content of 68%.

References

1. Klueber J, Costa J, Randow S, Codreanu-Morel F, Verhoeckx K, Bindslev-Jensen C, et al. Homologous tropomyosins from vertebrate and invertebrate: Recombinant calibrator proteins in functional biological assays for tropomyosin allergenicity assessment of novel animal foods. Clinical and Experimental Allergy. 2020 Jan 1;50(1):105.
2. Popp J, Trendelenburg V, Niggemann B, Randow S, Völker E, Vogel L, et al. Pea (Pisum sativum) allergy in children: Pis s 1 is an immunodominant major pea allergen and presents IgE binding sites with potential diagnostic value. Clin Exp Allergy. 2020 May 1;50(5):625–35.
3. Motoyama, K.; Suma, Y.; Ishizaki, S.; Nagashima, Y.; Shiomi, K. Molecular Cloning of Tropomyosins Identified as Allergens in Six Species of Crustaceans. 2007. Available online: https://pubs.acs.org/sharingguidelines (accessed on 3 July 2023).
4. Remillard, J.F.; Gould, M.C.; Roslansky, P.F.; Novitsky, T.J. Quantitation of endotoxin in products using the LAL kinetic turbidimetric assay. *Prog. Clin. Biol. Res.* **1987**, *231*, 197–210.
